# Interleukin-15 after Near-Infrared Photoimmunotherapy (NIR-PIT) Enhances T Cell Response against Syngeneic Mouse Tumors

**DOI:** 10.3390/cancers12092575

**Published:** 2020-09-10

**Authors:** Yasuhiro Maruoka, Aki Furusawa, Ryuhei Okada, Fuyuki Inagaki, Hiroaki Wakiyama, Takuya Kato, Tadanobu Nagaya, Peter L. Choyke, Hisataka Kobayashi

**Affiliations:** Molecular Imaging Program, Center for Cancer Research, National Cancer Institute, NIH, Bethesda, MD 20892, USA; ymaruoka@med.kyushu-u.ac.jp (Y.M.); Aki.Furusawa@nih.gov (A.F.); Ryuhei.Okada@nih.gov (R.O.); Fuyuki.Inagaki@nih.gov (F.I.); Hiroaki.Wakiyama@nih.gov (H.W.); Takuya.Kato@nih.gov (T.K.); nagaya@shinshu-u.ac.jp (T.N.); pchoyke@mail.nih.gov (P.L.C.)

**Keywords:** near infrared photoimmunotherapy, monoclonal antibodies, CD44, interleukin-15, cancer

## Abstract

**Simple Summary:**

Near infrared photoimmunotherapy is a newly developed and highly selective cancer treatment that employs a monoclonal antibody conjugated to a photo-absorber dye, IRDye700DX, which is activated by 690 nm light. Cancer cell-targeted near infrared photoimmunotherapy selectively induces rapid necrotic/immunogenic cell death only on target cancer cells and this induces antitumor host immunity including re-priming and proliferation of multi-chronal T-cells that can react with cancer-specific antigens. Interleukin-15 is a type-I cytokine that activates natural killer-, B- and T-cells while having minimal effect on regulatory T-cells that lack the interleukin-15 receptor. Therefore, interleukin-15 administration combined with cancer cell-targeted near infrared photoimmunotherapy could further inhibit tumor growth by increasing antitumor host immunity. In tumor-bearing immunocompetent mice receiving this combination therapy, significant tumor growth inhibition and prolonged survival was demonstrated compared with either single therapy alone, and tumor infiltrating CD8+ T-cells increased in number in combination-treated mice. Interleukin-15 enhances therapeutic effects of cancer-targeted near infrared photoimmunotherapy.

**Abstract:**

Near infrared photoimmunotherapy (NIR-PIT) is a newly developed and highly selective cancer treatment that employs a monoclonal antibody (mAb) conjugated to a photo-absorber dye, IRDye700DX, which is activated by 690 nm light. Cancer cell-targeted NIR-PIT induces rapid necrotic/immunogenic cell death (ICD) that induces antitumor host immunity including re-priming and proliferation of T cells. Interleukin-15 (IL-15) is a cytokine that activates natural killer (NK)-, B- and T-cells while having minimal effect on regulatory T cells (Tregs) that lack the IL-15 receptor. Here, we hypothesized that IL-15 administration with cancer cell-targeted NIR-PIT could further inhibit tumor growth by increasing antitumor host immunity. Three syngeneic mouse tumor models, MC38-luc, LL/2, and MOC1, underwent combined CD44-targeted NIR-PIT and short-term IL-15 administration with appropriate controls. Comparing with the single-agent therapy, the combination therapy of IL-15 after NIR-PIT inhibited tumor growth, prolonged survival, and increased tumor infiltrating CD8+ T cells more efficiently in tumor-bearing mice. IL-15 appears to enhance the therapeutic effect of cancer-targeted NIR-PIT.

## 1. Introduction

Near infrared photoimmunotherapy (NIR-PIT), which is a new type of cancer therapy that induces highly selective cell death on targeted cells only in NIR light exposed tumor beds, by employing a monoclonal antibody (mAb) conjugated to a silica-phthalocyanine photoabsorbing dye, (IRDye700DX: IR700) [1,2]. The antibody-IR700 conjugate is administered intravenously, followed by exposure to 690 nm NIR light which activates the IR700 dye. Tumor cells treated with NIR-PIT experience nearly immediate cell membrane damage and impaired cell function [3,4]. Treated cells show rapid volume expansion, followed by cell membrane rupture and extrusion of the cell contents into the extracellular space [3,5,6,7,8]. Unlike most other therapies that induce apoptotic cell death, NIR-PIT induces highly selective necrotic and immunogenic cell death of tumors with minimal damage to adjacent normal cells including immune cells in the tumor microenvironment (TME) [3,4]. NIR-PIT has shown to be effective clinically and a global phase III clinical trial of NIR-PIT using the EGFR-targeted antibody-IR700 conjugate, cetuximab-IR700 (ASP-1929) in patients with recurrent head and neck cancer is underway (https://clinicaltrials.gov/ct2/show/NCT03769506). Both the US Food and Drug Administration (FDA) and the Pharmaceuticals and Medical Devices Agency (PMDA) in Japan have given fast track status to this agent. 

In this study, we sought to augment the effects of tumor targeted NIR-PIT using interleukin-15 (IL-15). We employed CD44 as a tumor target, which is a well-known marker of cancer stem cells [9] and is expressed on the cell membrane of several cancers [10]. High expression of CD44 is associated with tumor aggressiveness, drug resistance, and poor treatment outcome [11,12]. NIR-PIT using anti-CD44-mAb-IR700 induces effective tumor killing in CD44-expressing syngeneic mouse models [13,14,15,16]. Cell membrane rupture after NIR-PIT releases tumor-specific antigens into the TME and promotes dendritic cell (DC) maturation, resulting in presentation of cancer-specific antigens on DCs to naive T cells for priming [14,15,17]. Thus, NIR-PIT causes both direct cell killing and indirect cell killing by antitumor immunity.

IL-15 has recently emerged as a candidate immune-activator for the treatment of cancer [18]. IL-15 induces adaptive immune responses that include T-cell proliferation, the generation of cytotoxic T lymphocytes (CTL), stimulation of immunoglobulin synthesis by B cells, and the generation and persistence of natural killer (NK) cells. Some of its functions are shared with IL-2 [19]. However, IL-15 is not required for the maintenance of regulatory T cells (Tregs) that suppress antitumor immune responses, unlike IL-2 [20]. Therefore, IL-15 can induce protective effects against cancer development with minimal effects on Tregs. In preclinical studies, the administration of IL-15 has antitumor effects in several mouse tumor models [21,22,23]. We hypothesized that IL-15 administration following cancer-antigen targeted NIR-PIT could enhance tumor growth inhibition. The purpose of this study was to investigate the in vivo therapeutic efficacy of CD44-targted NIR-PIT and IL-15 treatment in syngeneic mouse models of cancer compared to either therapy alone.

## 2. Results

### 2.1. Efficacy of CD44-Targeted NIR-PIT Combined with IL-15 Administration for MC38-luc Tumor

**Figure 1:**
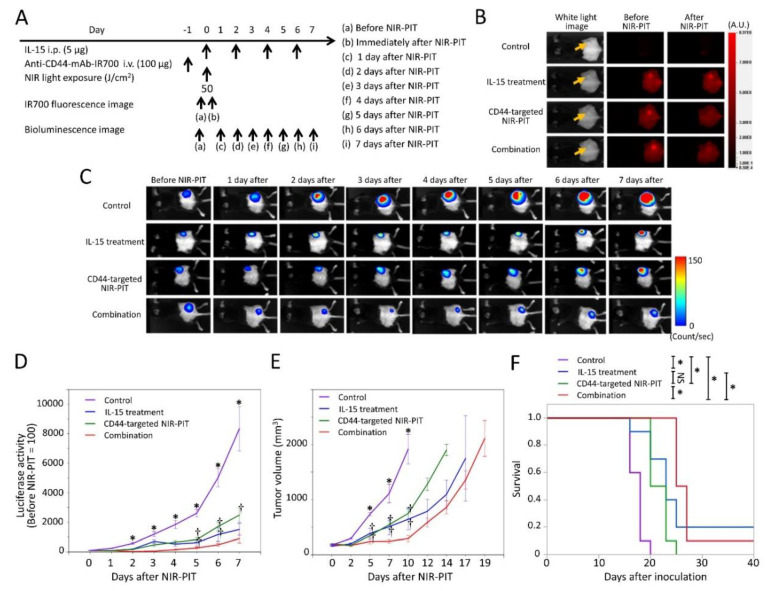
In vivo effect of CD44-targeted near infrared photoimmunotherapy (NIR-PIT) and interleukin-15 (IL-15) administration for MC38-luc tumor model. (**A**) NIR-PIT regimen. Bioluminescence and fluorescence images were obtained at each time point as indicated. i.p., intraperitoneal injection; i.v., intravenous injection. (**B**) Real-time in vivo IR700 fluorescence imaging of tumor-bearing mice before and approximately 10 min after NIR-PIT. The yellow arrows indicate the tumor locations. (**C**) In vivo bioluminescence imaging of tumor-bearing mice before and after treatment at the indicated timepoints. (**D**) Quantitative analysis of luciferase activity before and after treatment in tumor-bearing mice. *n* = 10/group, mean ± SEM; *, *p <* 0.05, vs. the other groups; †, *p <* 0.05, vs. combination group; Tukey–Kramer test. (**E**) Tumor growth in control and treated groups. *n* = 10/group, mean ± SEM; *, *p <* 0.05, vs. the other groups; †, *p <* 0.05, vs. combination group; Tukey–Kramer test. (**F**) Survival curves for control and treated groups. *n* = 10/group; *, *p <* 0.05; NS, not significant; log-rank test with Bonferroni correction.

In vitro and in vivo CD44 expression in all three tumor models (Figure S1) and cytotoxic effects of CD44 targeted NIR-PIT have been previously reported [24]. The NIR-PIT regimen and imaging protocol are depicted in Figure 1A. In the NIR-PIT treated group, 1 day after injection of anti-CD44-mAb-IR700, the tumors were exposed to 50 J/cm^2^ of NIR light on day 0. IR700 fluorescence signal in tumors decreased due to dispersion of fluorophore from dying cells and partial photo-bleaching in all cases (Figure 1B). To investigate tumor-killing efficacy after NIR-PIT, bioluminescence imaging was performed before and after treatment up to day 7 (Figure 1C). In most mice treated with NIR-PIT, luciferase activity decreased initially after NIR-PIT due to cell killing and then gradually increased as the tumor regrew (Figure 1C). In all treated groups luciferase activity was significantly lower at 2, 3, 4, 5, 6, and 7 days in the treatment groups than in the control group (*p <* 0.05, Tukey-Kramer test) (Figure 1D). CD44-targeted NIR-PIT combined with IL-15 treatment showed significantly lower luciferase activity compared with CD44-targeted NIR-PIT alone at 5, 6, and 7 days after NIR-PIT, with IL-15 (*p <* 0.05, Tukey–Kramer test) (Figure 1D). Tumor volume in all treated groups was significantly less at 5, 7, and 10 days compared with controls (*p <* 0.05, Tukey–Kramer test) (Figure 1E). The combination group showed significantly greater tumor reduction compared with IL-15 treatment or CD44-targeted NIR-PIT groups at 5, 7, and 10 days (*p <* 0.05, Tukey–Kramer test) (Figure 1E). These data demonstrate that CD44-targeted NIR-PIT combined with IL-15 resulted in the slowest rate of tumor regrowth compared with other groups. The combined therapy also was associated with significantly prolonged survival compared with IL-15 treatment alone or CD44-targeted NIR-PIT alone (*p <* 0.05, log-rank test) (Figure 1F). Thus, our results demonstrate that CD44-targeted NIR-PIT combined with IL-15 administration produced superior in vivo therapeutic results compared with the other two monotherapies for MC38-luc tumors.

### 2.2. Augmented CD8+ T Cells Infiltration in MC38-luc Tumors after CD44-Targeted NIR-PIT Combined with IL-15 Administration

**Figure 2:**
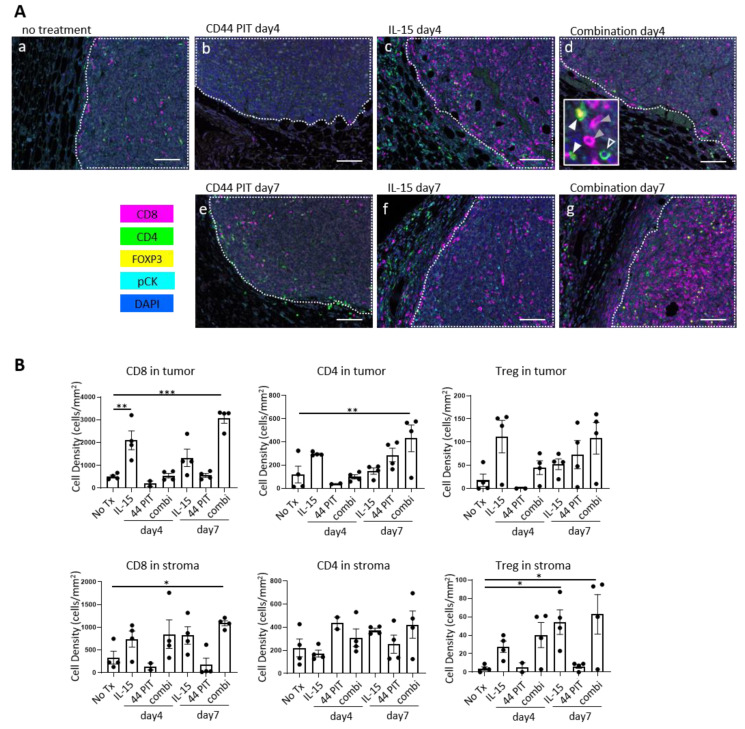
Immunohistochemical analysis after CD44-targeted NIR-PIT and/or IL-15 treatment in MC38-luc tumor model. (**A**) Representative multiplex immunohistochemistry (IHC) images in MC38-luc tumors 4 or 7 days after the treatments indicated. Antibody staining of CD8, CD4, FOXP3, and pan-Cytokeratin (pCK) are shown in magenta, green, yellow, and cyan respectively. Nucleus are stained with DAPI and shown in blue. Tumor areas are indicated in white dotted line. The inset in window d shows examples of CD8 T cell (gray filled arrowhead), CD4 T cell (open arrowhead), and Tregs (white filled arrowhead). Scale bar = 100 µm. (**B**) Cell density of CD8 T cells, CD4 T cells and Tregs in tumor and stroma calculated from IHC images. No Tx, no treatment; IL-15, IL-15 administration only; 44 PIT, CD44-targeted PIT only; combi, combination therapy of CD44-targeted PIT and IL-15 administration. Bars represent mean, dots represent individual samples, and error bars represent SEM. *n* = 4/group except *n* = 2 for CD44 day 4. **, *p <* 0.05, **, *p <* 0.01 ***, *p <* 0.0001; one-way ANOVA (followed by Dunnett’s multiple comparison test, vs. no Tx).

In order to assess the density of tumor infiltrating T cells, an important indicator of antitumor immune activity, multiplex immunohistochemistry (IHC) was performed in MC38-luc tumors (Figure 2, also see Figure S2). With IL-15 administration, the cell density of CD8 T cells within tumors significantly increased at day 4, however, this augmentation of CD8 T cells was less prominent at day 7, suggesting that the CD8 T-cell population seen at day 4 was short-lived. On the other hand, in the tumors treated with the combination therapy, the augmentation of CD8 T-cell infiltration was not observed at day 4. This result could be attributed to the cytotoxic effect of CD44-targeted PIT against activated immune cells which express CD44. However, the cell density of CD8 T cells inside the tumor tissue dramatically increased 7 days after the combination therapy. The infiltration of CD4 T cells also increased 7 days after the combination therapy. CD44 targeted NIR-PIT alone did not induce the increase of T cell population. These results indicated that only CD44-targeted NIR-PIT combined with IL-15 administration resulted in newly activated T cells within the tumor microenvironment.

The cell density of Tregs significantly increased in stroma 7 days after IL-15 administration and combination therapy. This was thought to be a secondary effect in response to the rapid increase of CD8 T cells.

We also tested the distribution of Granzyme B (Gzmb) expressing cytotoxic cells. With the combination of IL-15 and CD44-targeted NIR-PIT, the number of Gzmb-positive CD8 T cells in the tumor tissue significantly increased at day 7 after the therapy (Figure S3A,C). With IL-15 administration alone, we observed the increase of Gzmb-positive CD8 T cells was less significant. This result indicates that the combination therapy successfully increased the tumor-infiltration of fully differentiated effector T cells. In addition to CD8 T cells, after IL-15 administration or combination of IL-15 and CD44-targeted NIR-PIT, we also observed increased number of another Gzmb positive cytotoxic cells, which were not CD8- or CD4-positive, namely non-T cytotoxic cells (Figure S3A,B,D). Such non-T cytotoxic cells including NK cells were more prominent in day 4 than day 7, suggesting that IL-15 stimulates the cytotoxic activity of T and non-T cell population at acute and subacute phase of tumor rejection.

### 2.3. Efficacy of CD44-Targeted NIR-PIT Combined with IL-15 Administration for LL/2 Tumor 

**Figure 3:**
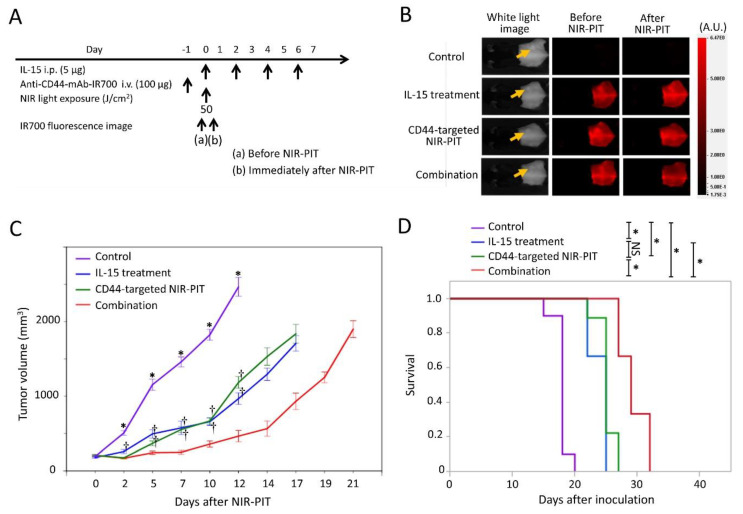
In vivo effect of CD44-targeted NIR-PIT and/or IL-15 administration in LL/2 tumor model. (**A**) NIR-PIT regimen. IR700 fluorescence images were obtained at each time point as indicated. i.p., intraperitoneal injection; i.v., intravenous injection. (**B**) Real-time in vivo IR700 fluorescence imaging of tumor-bearing mice before and approximately 10 min after NIR-PIT. The yellow arrows indicate the tumor locations. (**C**) Tumor growth in control and treated groups. *n* = 9/group, mean ± SEM; *, *p <* 0.05, vs. the other groups; †, *p <* 0.05, vs. combination group; Tukey–Kramer test. (**D**) Survival curves for control and treated groups. *n* = 9/group; *, *p <* 0.05; NS, not significant; log-rank test with Bonferroni correction.

**Figure 4:**
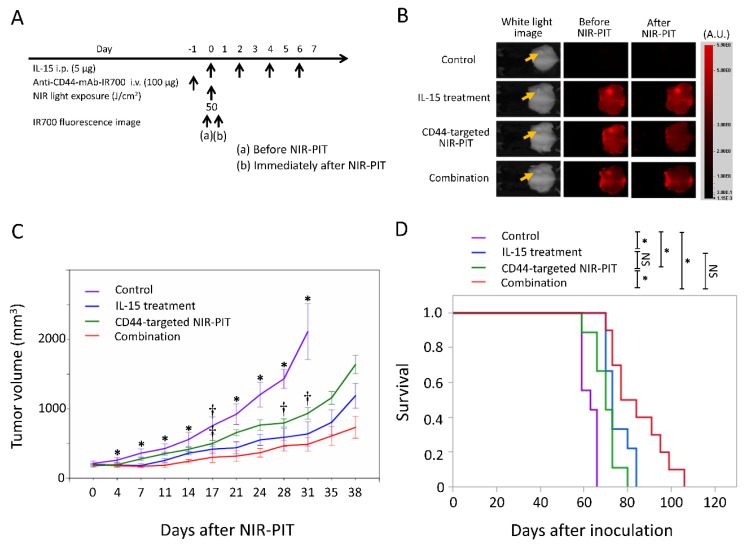
In vivo effect of CD44-targeted NIR-PIT and/or IL-15 administration in the MOC1 tumor model. (**A**) NIR-PIT regimen. IR700 fluorescence images were obtained at each time point as indicated. i.p., intraperitoneal injection; i.v., intravenous injection. (**B**) Real-time in vivo IR700 fluorescence imaging of tumor-bearing mice before and approximately 10 min after NIR-PIT. The yellow arrows indicate the tumor locations. (**C**) Tumor growth in control and treated groups. *n* = 10/group, mean ± SEM; *, *p <* 0.05, vs. the other groups; †, *p <* 0.05, vs. combination group; Tukey–Kramer test. (**D**) Survival curves for control and treated groups. *n* = 10/group; *, *p <* 0.05; NS, not significant; log-rank test with Bonferroni correction.

The NIR-PIT regimen and imaging protocol are depicted in Figure 3A. In the NIR-PIT treated group, 1 day after injection of anti-CD44-mAb-IR700, the tumors were exposed to 50 J/cm^2^ of NIR light on day 0. IR700 tumor fluorescence signal decreased due to dispersion of fluorophore from dying cells and partial photo-bleaching (Figure 3B). Tumor volumes in the treated groups were significantly reduced at all time points after treatment compared to the control group (*p <* 0.05, Tukey–Kramer test) (Figure 3C). The combination group showed significantly greater tumor reduction compared with the IL-15 treatment group at 2, 5, 7, 10, and 12 days after NIR-PIT (*p <* 0.05, Tukey–Kramer test) (Figure 3C), and showed significantly greater tumor reduction compared with CD44-targeted NIR-PIT group at 5, 7, 10, and 12 days after NIR-PIT (*p <* 0.05, Tukey–Kramer test) (Figure 3C). In the long-term follow-up, the combination group had significantly prolonged survival after NIR-PIT compared with IL-15 treatment or CD44-targeted NIR-PIT group (*p <* 0.05, log-rank test) (Figure 4D). This combined therapy was superior therapeutically to the other two types of monotherapies in LL/2 tumors.

### 2.4. Efficacy of CD44-Targeted NIR-PIT Combined with IL-15 Administration for MOC1 Tumor

Our previous study showed MOC1 tumor has low and more heterogeneous CD44 expression and CD44-targeted NIR-PIT was less effective against this type of tumor [13]. The NIR-PIT regimen and imaging protocol are depicted in Figure 4A. In the NIR-PIT treated group, 1 day after injection of anti-CD44-mAb-IR700, the tumors were exposed to 50 J/cm^2^ of NIR light on day 0. IR700 tumor fluorescence signal decreased due to dispersion of fluorophore from dying cells and partial photo-bleaching. (Figure 4B). The tumor volume in all treated groups was significantly reduced 4, 7, 11, 14, 21, 24, 28, and 31 days after NIR-PIT compared to the control group (*p <* 0.05, Tukey–Kramer test) (Figure 4C). The combination group showed significantly greater tumor reduction 17, 28, and 31 days after NIR-PIT compared to CD44-targeted NIR-PIT group (*p <* 0.05, Tukey–Kramer test). The combination therapy showed significantly prolonged survival compared to CD44-targeted NIR-PIT group, although IL-15 treatment group had no significant difference in survival compared to CD44-targeted NIR-PIT group (*p <* 0.05, log-rank test) (Figure 4D). This combined therapy was superior therapeutically to the other two types of single therapies in MOC1 tumors.

## 3. Discussion

The combination of IL-15 administration and CD44-targeted NIR-PIT showed superiority in survival to IL-15 treatment alone in all three tumor types tested here. IL-15 binds and sends signals through a heterotrimeric receptor composed of the IL-15-specific IL-15Rα, shared IL-2R/IL-15Rβ (CD122) with the IL-2 receptor, and the common type I cytokine receptor γ-chain (γc, CD132; [25,26]). IL-15 is highly bound through trans-presentation by IL-15Rα expressed on activated DCs or monocytes to the IL-2R/IL-15Rβ and γc on effector T, B, and NK cells [27]. When IL-15 binds its receptor, DCs can cause potent activation of the immune response [18,27]. Immunogenic cell death of cancer cells shortly after NIR-PIT releases damage-associated signals such as ATP, calreticulin, and high-mobility group box 1, which promotes DC maturation and subsequent effector T-cell activation mediated by IL-15 [3,14]. Thus, NIR-PIT and IL-15 administration trigger an immune response that is greater than either therapy alone. 

In all tumor types used in this study, MC38-luc, LL/2, and MOC1, infiltration of Tregs was observed within the TME [24]. Tregs tend to attenuate antitumor activity and are, therefore, not necessarily a desired outcome. However, IL-15 may be a safer choice of cytokine than IL-2 because the former does not stimulate Tregs while the latter is required for Treg maintenance [20]. In our study, significant increases in stromal Tregs were observed after IL-15 administration and the combination therapy, which was likely to be a secondary effect in response to the rapid increase of CD8 T cells in the TME. Our results showed that CD44-targeted NIR-PIT combined with short-term IL-15 treatment increased the infiltration of fully differentiated CD8+ effector T-cells which contributed to significantly prolonged survival compared to CD44-targeted NIR-PIT alone in all three types of tumors. Considering that administration of IL-15 alone only induced short-lived CD8 T cells, and CD44-targeted NIR-PIT alone did not increase the infiltration of T cells, the superior therapeutic effect of the combination therapy was thought to be the result of IL-15 mediated reinforcement of NIR-PIT-induced antitumor immune activation.

In addition to CD8 T cells, we observed increasing number of Gzmb-high non-T cytotoxic cells within a few days after IL-15 administration or the combination therapy. Since IL-15 is known to stimulate the cytotoxic function on both CD8 T and NK cells, these cells were most likely to be NK cells. Therefore, the combination therapy of CD44-targeted NIR-PIT and IL-15 administration demonstrate its therapeutic efficacy probably by stimulating both the NK-mediated innate immunity and T-cell mediated adoptive immunity.

This report represents an extension of previous studies which attempt to augment the immune response initiated by NIR-PIT. For instance, a previous study demonstrated that the combination of CD44-targeted NIR-PIT and either immune checkpoint blockade or Treg-targeted NIR-PIT showed inconsistent therapeutic efficacy among these three tumor models [13,14,24]. CD44-targeted NIR-PIT in combination with other therapies showed promising results in MC38-luc and LL/2 but was less effective against MOC1 tumor which has low CD44 expression and fewer CD44 positive tumor cells compared to MC38-luc and LL/2. On the other hand, in this study, the combination with IL-15 therapy was effective in all three tumor models including MOC1. Even though the release of damage-associated signals after NIR-PIT is likely to have been lower in MOC1 cells, IL-15 administration following NIR-PIT nevertheless contributed to enhancement of antitumor host immunity by activating tumor-attacking T and NK cells making this combination therapy similarly effective for all tumors. Further combination therapy of cancer-targeting NIR-PIT with both IL-15 and an immune-checkpoint blockade or Treg-targeting NIR-PIT could theoretically enhance tumor immunity. However, such therapies need to be designed cautiously since excessively enhanced immunity might induce cytokine storm as an adverse effect. Photodynamic therapy (PDT) is another therapeutic approach that utilizes light to treat cancer [28]. However, NIR-PIT is different from PDT in several aspects. Porphyrin-based photosensitizers used in conventional PDT do not selectively target cancer cells, resulting in damage to surrounding normal cells in organs or vasculatures. In contrast, because NIR-PIT induces selective immunogenic cell death only on targeted cancer cells in NIR light exposing tumor beds, it spares the normal cells including all immune cells in the TME allowing them to play a role in immune activation [3]. Therefore, rapid and effective activation of antitumor host immunity is induced by NIR-PIT [14], whereas such effects are not generally induced by nonselective cell killing with PDT.

This study had several limitations. First, we used subcutaneously implanted tumor models, whereas an orthotopic or transgenic mouse model would be considered as a superior clinically relevant animal model [29]. However, in the present study, it was technically important to obtain a consistent size, shape, and location of each tumor in order to fairly compare tumor growth between tested groups. The orthotopic model could produce variable results depending on surgical procedure for implanting tumor within the organ. Second, although CD44 is expressed on these syngeneic cancer cells, CD44 is also expressed on activated immune cells. CD44-targeted NIR-PIT could thus damage effector immune cells. Indeed, counts of all kinds of T cells decreased 4 days after CD44-targeted NIR-PIT alone. Thus, CD44 is not ideal as a tumor target. Since NIR-PIT is applicable to any antibody that targets tumor cell surface antigens [30], NIR-PIT using an anti-mouse EGFR antibody, the target molecule in NIR-PIT clinical trials, would have been preferable. However, there is no thoroughly established EGFR+ mouse tumor model, and commercially available anti-mouse EGFR Abs are too expensive for experimental cancer therapy studies. Further investigation is required to clarify the degree of enhancement of antitumor host immunity through NIR-PIT. Third, we may not have optimized the dosing of IL-15. IL-15 has a circulation half-life of around 1 h [31] that is longer than IL-2 (15 min), and only 5 μg of IL-15 was administered i.p. up to 1 week after CD44-targeted NIR-PIT. A more prolonged administration of IL-15 might have induced greater therapeutic effects albeit balanced by an increased risk of lymphoproliferative disorder or lymphoma, either of which was seen in the IL-15 transgenic mouse [32]. In a previous article, Yu et al. reported that administration of IL-15 alone simultaneously activated both immune-system and negative regulatory checkpoints that might weaken the antitumor immune response [22]. To elucidate the reason why the treatment regimen of this combined NIR-PIT therapy with IL-15 was difficult to cure tumors, further investigation of the T cell status such as exhaustion, activation, and memory differentiation would be required.

## 4. Materials and Methods

### 4.1. Cell Culture

MC38 cells (murine colon cancer), which were generously provided by Dr. Thomas Waldmann, NIH [33] stably expressing luciferase (MC38-luc, generated via stable transduction with RediFect Red-Fluc lentivirus from PerkinElmer per manufacturer recommendations), which was purchased from ATCC (Rockville, MD, USA), LL/2 cells (murine Lewis lung carcinoma), which was purchased from ATCC (Rockville, MD, USA), and MOC1 cells (murine oral carcinoma), which were produced and generously provided by Dr. Clint Allen, NIH [34] were used in this study. Luciferase expression on the MC38-luc cells was confirmed through 10 passages. MC38-luc and LL/2 cells were cultured in RPMI1640 supplemented with 10% FBS and 1% penicillin–streptomycin (all Gibco brand, ThermoFisher Scientific, Waltman, MA, USA) in tissue culture flasks (182 cm^2^; CELLTREAT Scientific Products, Pepperell, MA, USA) in a humidified incubator at 37 °C in an atmosphere of 95% air and 5% carbon dioxide. MOC1 cells were cultured in HyClone Iscove’s modified Dulbecco’s medium (Crytiva, Marlborough, MA, USA)/HyClone Ham’s Nutrient Mixture F12 (Crytiva, Marlborough, MA, USA) at a 2:1 mixture with 5% FBS, 1% penicillin/streptomycin, 3.5 ng/mL EGF (MilliporeSigma, Burlington, MA, USA), 40 ng/mL hydrocortisone (MilliporeSigma, Burlington, MA, USA), and 5 ng/mL insulin (MilliporeSigma, Burlington, MA, USA) in the tissue culture flasks in a humidified incubator at 37 °C in an atmosphere of 95% air and 5% carbon dioxide. Cells were authenticated via in vitro growth characteristics.

### 4.2. Reagents

Water soluble, silica-phthalocyanine derivative, IRDye700DX NHS ester was obtained from LI-COR Bioscience (Lincoln, NE, USA). An anti-mouse/human CD44 mAb (IM7) was purchased from Bio X Cell (Lebanon, NH, USA). All other chemicals were of reagent grade.

### 4.3. Synthesis of IR700-Conjugated Anti-CD44 mAb 

Briefly, anti-CD44-IgG (1 mg, 6.7 nmol/L) was incubated with IR700 NHS ester (65.1 μg, 33.3 nmol, 10 mmol/L in DMSO) and 0.1 mol/L Na2HPO4 (pH 8.5) at room temperature for 1 h. The mixture was purified with a gel filtration column (Sephadex G 25 column, PD-10, Crytiva, Marlborough, MA, USA). The protein concentration was measured with Coomassie Plus protein assay kit (Thermo Fisher Scientific Inc, Waltman, MA, USA) by determining absorption at 595 nm with a UV-Vis spectroscopy system (8453 Value System; Agilent Technologies, Santa Clara, CA, USA). We abbreviate IR700-conjugated anti-CD44 mAb as anti-CD44-mAb-IR700.

### 4.4. Animal Model 

All procedures were performed in compliance with the Guide for the Care and Use of Laboratory Animals and approved by the local Animal Care and Use Committee on 12-02-2019 (MIP-003). Six- to eight-week-old female C57BL/6 mice (strain #000664) were purchased from the Jackson laboratory (Bar Harbor, ME, USA). The lower part of the body of the mice was shaved as fur can interfere with light activation of the conjugate and bioluminescence imaging. Mice with tumors reaching approximately 150 mm^3^ in volume were used for the experiments. Tumor volumes were calculated from the greatest longitudinal diameter (length) and the greatest transverse diameter (width) using the following formula; tumor volume = length × width^2^ × 0.5, based on caliper measurements. Mice were monitored each day and tumor volumes were measured three times a week for MC38-luc and LL/2 tumors and twice a week for MOC1 tumors until the tumor volume reached 2000 mm^3^, whereupon the mice were euthanized by inhalation of carbon dioxide gas. 

### 4.5. In Vivo NIR-PIT

MC38-luc cells (8 million), LL/2 cells (8 million), and MOC1 cells (4 million) were subcutaneously injected in the dorsum of mice. Once tumors reached volumes of approximately 150 mm^3^ mice were divided randomly into one of the following four experimental groups: (1) no treatment (control); (2) intraperitoneal injection of 5 μg murine IL-15 (PeproTech, Rocky Hill, NJ, USA) on days 0, 2, 4, and 6 following intravenous injection of 100 μg anti-CD44-mAb-IR700 (IL-15 treatment); (3) intravenous injection of 100 μg anti-CD44-mAb-IR700 followed by external NIR light irradiation at 50 J/cm^2^ on day 0 (CD44-targeted NIR-PIT); (4) intravenous injection of 100 μg anti-CD44-mAb-IR700 followed by external NIR light irradiation at 50 J/cm^2^ on day 0 and 100 J/cm^2^ on day 1 with intraperitoneal injection of 5 μg murine IL-15 on days 0, 2, 4, and 6 (combination). For the mice with MC38-luc tumor, LL/2 tumor, and MOC1 tumor in the NIR-PIT treated groups, intravenous injection of the anti-CD44-mAb-IR700 was performed 5, 5, and 28 days after tumor inoculation, respectively. NIR light was administered to tumor-bearing mice using a red-light emitting diode (LED), which emits light in the range of 670–710 nm wavelength (L690-66-60; Marubeni America Co., New York, NY, USA) at a power density of 50 mW/cm^2^ as measured with an optical power meter (PM 100, Thorlabs, Newton, NJ, USA). IR700 absorbs light at approximately 690 nm. IR700 fluorescence images were obtained before and after therapy.

### 4.6. In Vivo Bioluminescence Imaging and IR700 Fluorescence Imaging

To obtain bioluminescence images in MC38-luc tumor-bearing mice, D-luciferin (15 mg/mL, 150 mL; Goldbio, St Louis, MO, USA) was intraperitoneally injected. Luciferase activity was analyzed with a bioluminescence imaging system (Photon Imager; Biospace Lab, Nesles la Vallée, FRANCE) using units of relative light units (RLU). Regions of interests (ROIs) were placed over the entire tumor. The counts per minute of RLUs were calculated using M3 Vision Software (Biospace Lab) and converted to a percentage ratio based on the following formula: [(RLU after treatment)**/**(RLU before treatment) × 100 (%); [35]]. Bioluminescence imaging was performed before and after NIR-PIT (protocol below) on day 0 to day 7. In vivo IR700 fluorescence images were obtained with a Pearl Imager (LI-COR Biosciences, Lincoln, NE, USA) using the 700-nm fluorescence channel.

### 4.7. Multicolor Immunofluorescence

Multicolor immunofluorescence was performed using Opal 7-Color Automation immunohistochemistry staining Kit (Akoya Bioscience, Menlo Park, CA, USA) and BOND RXm auto stainer (Leica Biosystems, Wetzlar, Germany). The following antibodies were used: anti-CD44 (clone IM7; 1:5000 dilution; Bio X Cell, Lebanon, NH, USA), anti-CD8 (clone EPR20305; 1:500 dilution; Abcam, Cambridge, MA, USA), anti-CD4 (clone EPR19514; 1:1000 dilution; Abcam), anti-FOXP3 (clone 1054C; 1:1000 dilution; Novus Biologicals, Littleton, CO, USA), anti-pan cytokeratin (rabbit poly; 1:500 dilution, Bioss, Woburn, MA, USA), anti-CD45 (clone D3F8Q, 1:500 dilution, Cell Signaling Technology, Danvers, MA, USA), and anti-Granzyme B (rabbit poly; 1:500 dilution, Abcam). The staining was performed according to the Opal 7 color protocol provided by manufacturer with following modification: (i) antigen retrieval was performed using BOND ER2 solution (Leica Biosystems, Wetzlar, Germany) for 20 min and (ii) the ImmPRESS HRP anti-Rabbit IgG (Peroxidase) Polymer Detection Kit (Vector Laboratories, Burlingame, CA, USA) was used instead of anti-mouse/human secondary antibody provided in the kit. Stained slides were mounted with VECTASHIELD Hardset Antifade Mounting Medium (Vector Laboratories) and then imaged using Mantra Quantitative Pathology Workstation (Akoya Biosciences). Images were analyzed with inForm software (Akoya Biosciences). inForm software was trained to detect tissues and cell phenotypes according to following criteria: areas with pan-cytokeratin expression = tumor, other areas = stroma, CD4+FOXP3+ cells = Tregs, CD4+FOXP3− = CD4 T cells, CD8+ = CD8+ T cells, respectively. At least five images were taken from each tumor sample, areas for each tissue phenotype and cell count of each phenotype were combined to calculate the cell density. As for Gzmb expression analysis, cell phenotyping (CD8+/CD45+ = CD8+ T cells, CD4+/CD45+ = CD4+ T cells, Gzmb+/CD8−/CD4− = non-T-cell cytotoxic cells) and Gzmb expression analysis (Gzmb + and other) were performed separately using inForm for the same set of images, then cell segmentation data were consolidated and analyzed using phenoptrReports and phenoptr (Akoya Biosciences) to calculate the density of Gzmb+CD8 T cells.

### 4.8. Statistical Analysis

Quantitative data were expressed as means ± SEM. The Mann–Whitney U test was used to compare differences between two groups. For multiple comparisons (≥3 groups), a one-way analysis of variance (ANOVA) followed by the Tukey–Kramer test or Dunnett’s multiple comparison test was used. The cumulative survival was analyzed by the Kaplan–Meier survival curve analysis, and the differences between tested groups were compared using the log-rank test followed by Bonferroni correction. Statistical analysis was performed with JMP 13 software (SAS Institute, Cary, NC, USA). A *p* value of less than 0.05 was granted significant.

## 5. Conclusions

CD44-targeted NIR-PIT combined with IL-15 treatment showed superior in vivo therapeutic efficacy to either CD44-targeted NIR-PIT or IL-15 treatment alone in colon, lung, and oral cancer models. IL-15 might have potential to become a versatile adjuvant after cancer NIR-PIT.

## Supplementary Materials

The following are available online at https://www.mdpi.com/2072-6694/12/9/2575/s1, Figure S1: CD44 expression within MC38-luc (A), LL/2 (B), and MOC1 (C) tumors, Figure S2: Tissue and cell phenotyping based on the multiplex immunohistochemistry (IHC), Figure S3: Distribution of Granzyme B (Gzmb) expressing cytotoxic cells within the treated tumors.
