# Supplementary material for: Interleukin-15 after Near-Infrared Photoimmunotherapy (NIR-PIT) Enhances T Cell Response against Syngeneic Mouse Tumors
Source: Cancers (Basel). 2020 Sep 10;12(9):2575. doi: 10.3390/cancers12092575 (PMC7564397; doi:10.3390/cancers12092575)
Supplement: Supplementary file 1 [file cancers-12-02575-s001.pdf]

Supplemental information

Interleukin-15 after near-infrared photoimmunotherapy (NIR-PIT) enhances T cell response against syngeneic mouse tumors

Yasuhiro Maruoka[1], Aki Furusawa[1], Ryuhei Okada[1], Fuyuki Inagaki[1], Hiroaki Wakiyama[1], Takuya Kato[1], Tadanobu Nagaya[1], Peter L. Choyke[1], Hisataka Kobayashi[1]

[1]Molecular Imaging Program, Center for Cancer Research, National Cancer Institute, NIH, Bethesda, MD, 20892, USA

Running title: IL-15 after NIR-PIT enhances anti-tumor immunity

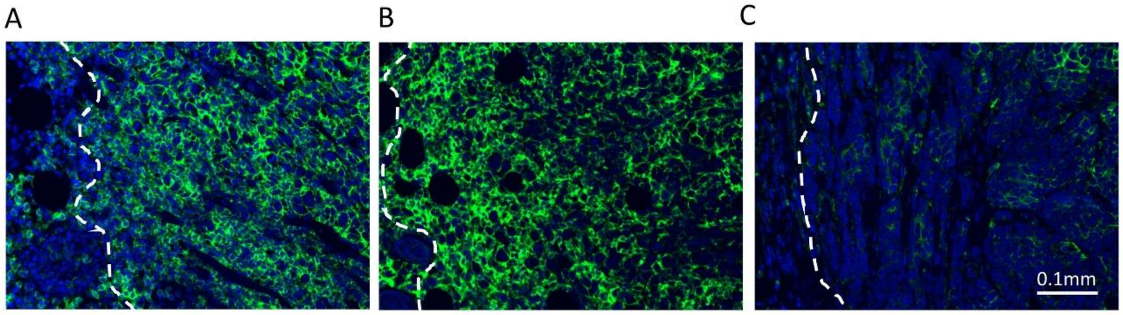

Figure S1. CD44 expression within MC38-luc (**A**) LL/2 (**B**) and MOC1 (**C**) tumors. Immunohistochemistry staining was performed to examine CD44 expression shown in green. Cell nuclei are stained with DAPI shown in blue.

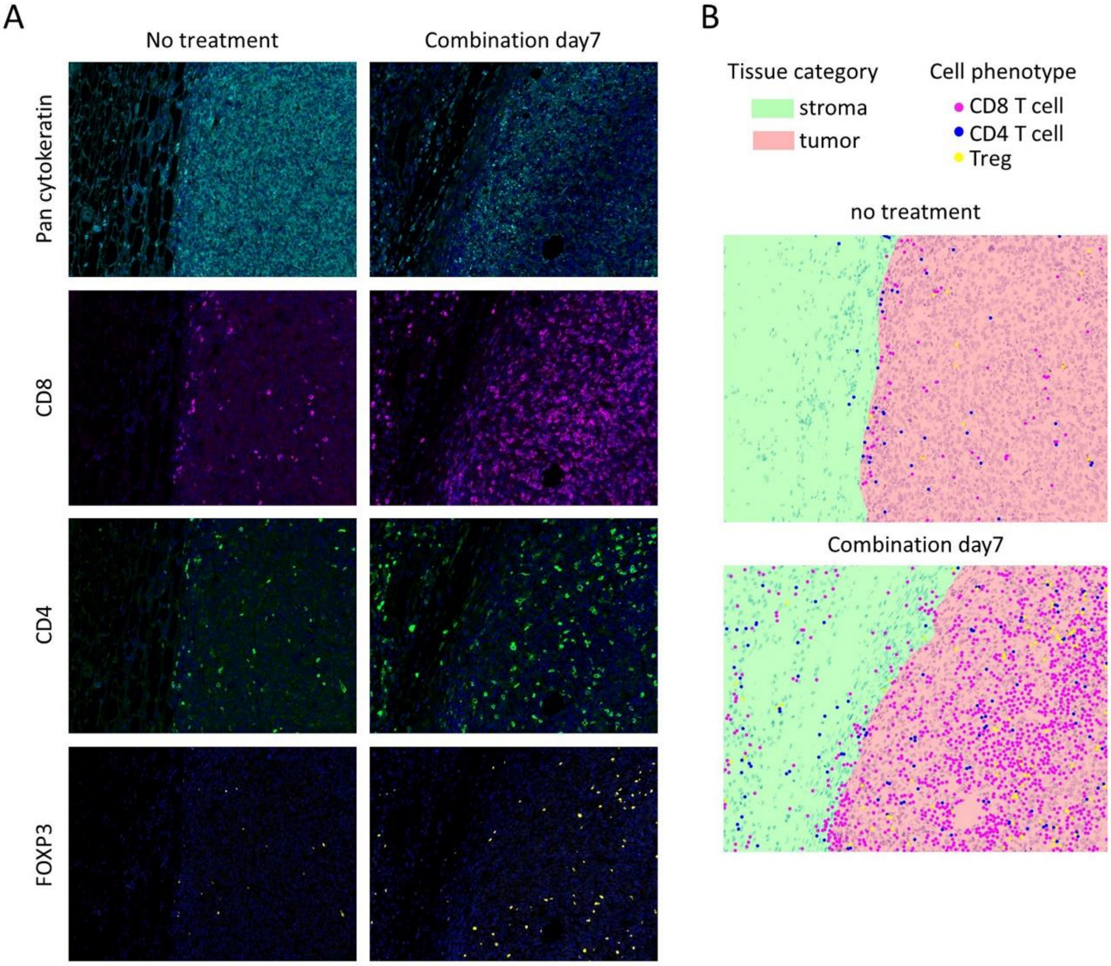

Figure S2. Tissue and cell phenotyping based on the multiplex immunohistochemistry (IHC). (**A**) single channel + DAPI images from the composite images shown in figure 2A. (**B**) tissue and cell phenotyping based on the IHC shown in A. tumor tissue (pan-cytokeratin+) is shown in pink and stroma is shown in green. Magenta, blue and yellow dots represent CD8 T cell (CD8+), CD4 T cell (CD4+/FOXP3−) and Tregs (CD4+/FOXP3+) respectively.

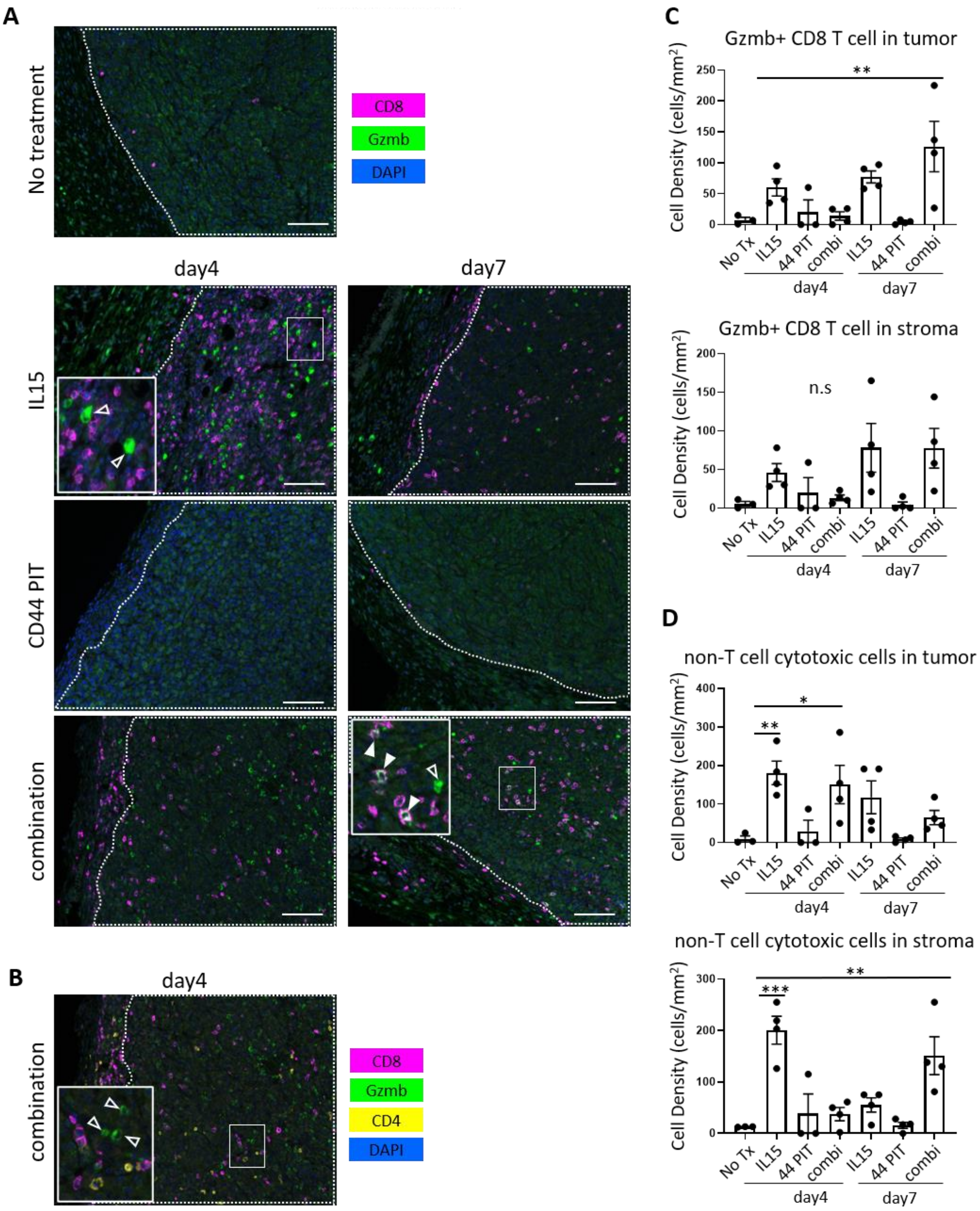

Figure S3. Distribution of Granzyme B (Gzmb) expressing cytotoxic cells within the treated tumors. (**A**) multicolor IHC images of tumors 4 and 7 days after IL-15 administration, CD44-targeted NIR-PIT and combination therapy. CD8 expression and Gzmb expressions are shown in magenta and green respectively. Tumor areas are enclosed with dotted white line. Insets show enlarged images of the area enclosed in rectangle in each picture. Examples of fully differentiated cytotoxic T cells (CD8+/Gzmb+) and non-T-cell cytotoxic cells (CD8-/Gzmb+) are indicated with white filled arrowheads and open arrowheads respectively. Scale bar = 100 µm. (**B**) multicolor IHC images of day 4 after combination therapy shown in A with CD4 expression (yellow) added. Note that there are Gzmb high cells which do not

overwrap with CD8 nor CD4. (**C**) Cell density of fully differentiated cytotoxic T cells (CD8+/Gzmb+) shown as cell count per mm^2 area in stroma and tumor. n=3 or 4; **, p<0.01 (one-way ANOVA followed by Dunnett's multiple comparison test vs no Tx.) (**D**) Cell density of non-T-cell cytotoxic cells (CD8-/CD4-/Gzmb+) cells per area in stroma and tumor. n=3 or 4; *, p<0.05; **, p<0.01; ***, p<0.0001 (one-way ANOVA followed by Dunnett's multiple comparison test, vs no Tx).
